# Raman and Fluorescence Enhancement Approaches in Graphene-Based Platforms for Optical Sensing and Imaging

**DOI:** 10.3390/nano11030644

**Published:** 2021-03-05

**Authors:** Sandra Cortijo-Campos, Rafael Ramírez-Jiménez, Alicia de Andrés

**Affiliations:** 1 Instituto de Ciencia de Materiales de Madrid, Consejo Superior de Investigaciones Científicas, Cantoblanco, 28049 Madrid, Spain; s.cortijo@csic.es (S.C.-C.); ramirez@fis.uc3m.es (R.R.-J.); 2 Departamento de Física, Escuela Politécnica Superior, Universidad Carlos III de Madrid, Avenida Universidad 30, Leganés, 28911 Madrid, Spain

**Keywords:** nanoparticles, graphene, plasmonics, SERS, interference, enhanced Raman scattering, enhanced fluorescence, FRET, resonant Raman scattering, graphene nanodots, optical simulations

## Abstract

The search for novel platforms and metamaterials for the enhancement of optical and particularly Raman signals is still an objective since optical techniques offer affordable, noninvasive methods with high spatial resolution and penetration depth adequate to detect and image a large variety of systems, from 2D materials to molecules in complex media and tissues. Definitely, plasmonic materials produce the most efficient enhancement through the surface-enhanced Raman scattering (SERS) process, allowing single-molecule detection, and are the most studied ones. Here we focus on less explored aspects of SERS such as the role of the inter-nanoparticle (NP) distance and the ultra-small NP size limit (down to a few nm) and on novel approaches involving graphene and graphene-related materials. The issues on reproducibility and homogeneity for the quantification of the probe molecules will also be discussed. Other light enhancement mechanisms, in particular resonant and interference Raman scatterings, as well as the platforms that allow combining several of them, are presented in this review with a special focus on the possibilities that graphene offers for the design and fabrication of novel architectures. Recent fluorescence enhancement platforms and strategies, so important for bio-detection and imaging, are reviewed as well as the relevance of graphene oxide and graphene/carbon nanodots in the field.

## 1. Introduction 

**Figure 1:**
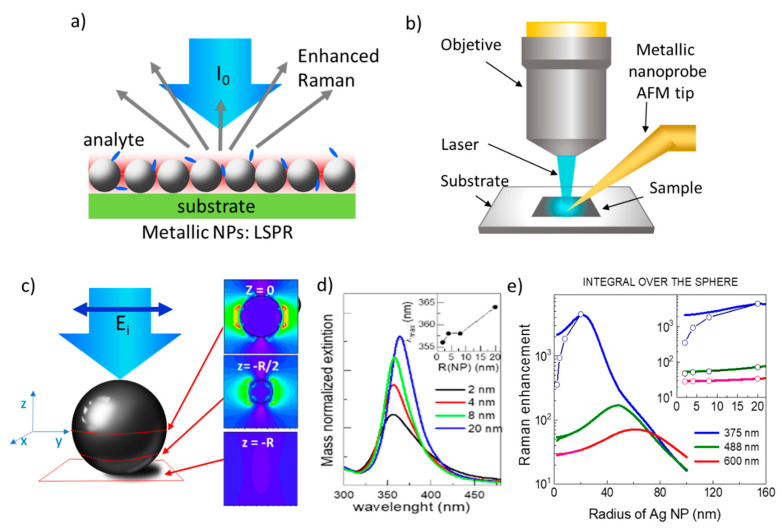
(**a**) Schema of the surface-enhanced Raman scattering (SERS) effect originated by the localized plasmon resonance of metallic inter-nanoparticles (NPs); (**b**) tip-enhanced Raman spectroscopy where a nanosized metal tip is used to increase the Raman signal and the spatial resolution to the nm scale; (**c**) inhomogeneous induced electric field at the NPs by the incident radiation with the polarization indicated by the blue arrow; (**d**) Mie calculations of extinction vs. Ag NP radius in the ultra-small limit normalized to the NP mass with corrected dielectric constant. Inset: plasmon resonance wavelength vs. NP radius (adapted with permissions from [32]); (**e**) calculated integral over the whole NP surface of the Raman enhancement as a function of the NP radius and incident laser wavelength. The inset shows the effect of considering the damping.

Optical spectroscopies are commonly used to detect a wide range of analytes of different nature, either sensing a fluorescence signal of the molecule itself or of a linked fluorophore or directly detecting its specific Raman vibration modes. Fluorescence often allows very low detection limits but still, an increased sensitivity is needed, for example, to detect the fluorescent DNA binding dyes used for the analysis and quantification of nucleic acids through real-time polymerase chain reaction (PCR) [1]. However, fluorescence detection usually requires exogenous markers, and, in some cases, the autofluorescence from the sample itself can be an important drawback. Raman spectroscopy directly enables the identification of a specific species among different components since the vibrational spectrum is a molecule fingerprint, however, its cross-section is very low, about 10^10^ times lower than vibrational absorption, hindering low concentration detection. These optical techniques present several important characteristics for detection, identification, and imaging, such as being cost-effective, rapid, non-destructive, and having sub-micron resolution, reaching <10 nm with tip-enhanced Raman spectroscopy (TERS) [2], (Figure 1b) and quite good penetration depth in biological materials which can be enhanced by looking at the spectral regions within the so-called biological windows, in the IR. Therefore, since the observation of the enhancement of pyridine Raman spectrum on roughened silver electrodes in 1977 [3], great efforts have been underway to increase Raman signal intensity, mainly, but not only, through the coupling to localized surface plasmon resonances (LPSR) of metallic nanoparticles, called SERS (surface-enhanced Raman scattering) (Figure 1a) [4,5,6,7]. SERS can detect a wide variety of chemical species such as toxic or radioactive cations/anions, ionic nutrients [8], pesticides [9], drugs and pharmaceuticals, or explosive materials [10]. The highest enhancements are obtained when the molecule is located at hot-spots, occurring for example at small gaps in between two very close nanoparticles (NPs) or at sharp tips of NPs with particular shapes [11]. Therefore, obtaining, controlling, and simulating hot-spot formation is a crucial point. Besides this electromagnetic enhancement mechanism (EM), a chemical mechanism (CM) occurs in the SERS amplification process that was estimated to be limited to up to 100 fold [12]. However, recently, a strong focus has been put on the relevance of the role of chemical coupling and capture of molecules to further enhance amplification [13]. In this review, we will only focus on less revised aspects of SERS, specifically on the role of the inter-particle distance to generate electric field hot-spots and on the possible interest of few-nanometer-sized nanoparticles.

Nanostructured metals have been shown to also enhance fluorescence in the visible range with amplifications in the order of 10-fold for silver NPs [14], and more recently, also in the UV range using aluminum [15], but the mechanism of the metal-enhanced fluorescence (MEF) is still not well understood. SERS and MEF have many common points but actually are usually seen as quite separated fields. Here, metal NP-related enhancements in both Raman and fluorescence processes are tackled. However, beyond the search for increasing enhancement factors (EF), several other issues are important. The platforms should also allow the quantification of the molecule concentration or to obtain images thus requiring stability and reproducibility and, especially for the quantification, efficiently solving the difficult task of precisely evaluating the EF and controlling its homogeneity across the platform. In this context, graphene has a relevant role to play. Including graphene and graphene-related materials in platforms for optical sensing offers not only increased or new functionalities but also facilitates the fabrication of novel architectures. This review collects different approaches based on hybrid systems that include graphene-related materials with the purpose of increasing Raman and fluorescence signals to facilitate molecule identification and detection and also to increase the quality of optical images of organic and inorganic systems.

The first developed approach has been the combination of noble metal NPs with graphene to improve the performance and stability of plasmonic sensing systems [16,17,18]. The role of graphene is analyzed here. Graphene is polyvalent, serving as a substrate, a building block, a Raman probe, displaying as well an adequate chemical compatibility with most molecules and acting as an averaging medium of the strongly spatially variable SERS effect. Moreover, graphene can also provide an extra enhancement of a chemical nature, called “graphene-enhanced Raman scattering” (GERS), that strongly depends on the interaction between the molecules and graphene [17,19]. Graphene oxide (GO) and reduced GO with different reduction degrees are especially adequate for establishing covalent bonds and thus of interest in biosensing. More recently single and few-layer graphene nanodots, as well as carbon nanodots that present adequate and tunable photoluminescence (PL) properties, have been found to be promising components, especially in fluorescence detection and imaging. Indeed, graphene derivatives are utilized in “theranostics” of cancer, which refers to the ability to simultaneously or sequentially diagnose and treat diseases [20]. However, their biocompatibility is under study and proof-of-concept investigations in cancer therapy are still in the pre-clinical phase [21].

This review also focuses on alternative enhancement mechanisms for optical signals and collects cases where several mechanisms can be combined. One option to increase Raman signal is by tuning the excitation laser energy with that of real electronic transitions of the molecules to be detected, taking advantage of the resonant Raman scattering (RRS) process. A recent focus in this area is the search of SERS amplification platforms efficient in the UV, to combine SERS and RRS, based on several metals that present plasmon resonances in the UV, such as aluminum [22,23] or rhodium [24], but with much lower efficiency than that of silver or gold in the visible range [25]. Another enhancement mechanism that is reviewed here and which has been scarcely explored for Raman spectroscopy is related to the amplification of an electromagnetic signal based on interference processes. Multilayered heterostructures based on thin films of materials can be designed and fabricated to optimize the interference of light occurring at the interfaces and can be tuned depending on the application and the excitation wavelength. The interference-enhanced Raman scattering (IERS) was defined in the 1980s and was initially used to detect the phonons of inorganic ultra-thin films [26,27]. Since the discovery of the properties of exfoliated graphene, single-crystalline silicon wafers with a SiO_2_ layer, typically hundreds of nanometers thick, are commonly used to better visualize and differentiate graphene flakes with a different number of layers. Now, Si/SiO_2_ substrates are used for this purpose to increase the Raman signal of 2D (or few-layer) materials with Raman gains up to around 40 [28,29,30,31].

The following section of this review (Section 2) briefly collects relevant information on SERS from non-interacting metallic NPs and focuses on the relevance of inter-NP distance and on the ultra-small NP limit. Section 3 collects the role of graphene and graphene oxide in systems that combine metallic NPs with graphene for Raman enhancement. Section 4 summarizes the recent investigations on Raman enhancement by light interference and Section 5 is devoted to the amplification of fluorescence signals and to increasing the contrast in optical images. The mechanism of metal-enhanced fluorescence is outlined as well as the different roles that graphene-related materials (graphene oxide and graphene and carbon nanodots) play in detection and imaging.

## 2. SERS in Noble Metal NPs

The literature on SERS is massive and excellent reviews have been published both on experimental and theoretical aspects (see for example [4,5,6,19,33]). Here we collect some general conclusions regarding the relevance of size and shape on the amplification capabilities and focus on less explored aspects which are the inter-NP distance and the ultra-small NP size limit.

Local surface plasmon resonances (LSPR) occur in highly polarizable objects, such as nanosized metallic particles, that produce intense localized electric fields for specific incident light energies and originate huge enhancements of the Raman signals of vibration modes of molecules or phonons of solids when these are located in the NPs’ close vicinity (Figure 1a). In a first approximation, the LSPR electric field amplitude, *E,* decays as a dipole field, *E* ~ *d_m-NP_*^−3^, where *d_m-NP_* is the molecule-NP distance [4,34,35]. Experimentally, the optimum size of non-interacting spherical NPs is found to be in the range of 40–100 nm for gold and silver [36,37] but NPs’s optical properties strongly depend on their size, shape, and composition so that there is an intense activity to optimize these parameters [38,39,40,41].

Analytical calculations of optical extinction (absorption and scattering) can be obtained based on the Mie theory [42] which is exact for isolated spherical particles since it is a solution to Maxwell equations of plane waves impinging on metal/dielectric isolated spheres. In the case of non-spherical objects, the solution is much more complicated. In this case, it is possible to get an intuitive picture of the extinction process with non-interacting NPs using the dipolar approximation when the NP size is much smaller than the wavelength. For larger NPs multipolar terms should be taken into account together with electrodynamic corrections: the effect of the radiated field by induced dipoles. For metal NPs of very small sizes, surface damping plays an important role as the electron mean free path begins to approach the size of the nanoparticle, thus additional damping needs to be considered, in any of these approaches, by modifying the dielectric constant of the particle [32,33,34]. The extinction band correlates with the near-field electric field which originates the Raman enhancement.

Mie calculations for Ag NPs, show that the plasmon resonance as a function of NP diameter is red-shifted as the size increases, slightly (~8 nm) for NPs from 4 to 40 nm (Figure 1c) and more importantly for diameters > 40 nm with a significant widening and damping for these large NPs [38]. The most efficient plasmon for non-interacting Ag particles is calculated to be around 40 nm (diameter) however, it is located in the UV (~365 nm) which is not convenient for detection with Raman systems that use visible excitation. The LSPR spatial distribution of one NP is highly inhomogeneous and depends on the incident light polarization (Figure 1c), therefore, to obtain the averaged Raman amplification of n NP, which is the relevant value when the quantitative determination of molecule concentration is searched, it is necessary to integrate over the whole surface of the NP. Figure 1e shows such integrated Raman amplification using the dipolar approximation for the Mie theory, as a function of the NP radius for different incident excitation wavelengths. Indeed, the maximum amplification is obtained for NPs around 20 nm radius (40 nm diameter) when using laser excitation at 375 nm, but shifts to larger sizes for visible excitation wavelengths. The open circles show the results considering the damping effect for the small NP size end. The importance of the damping at the NP surface in the small limit is also visualized in the reduction of the resonance intensity when normalizing to the NP mass especially for radii below around 10 nm (Figure 1d) hampering SERS applications for the smallest non-interacting NPs.

**Figure 2:**
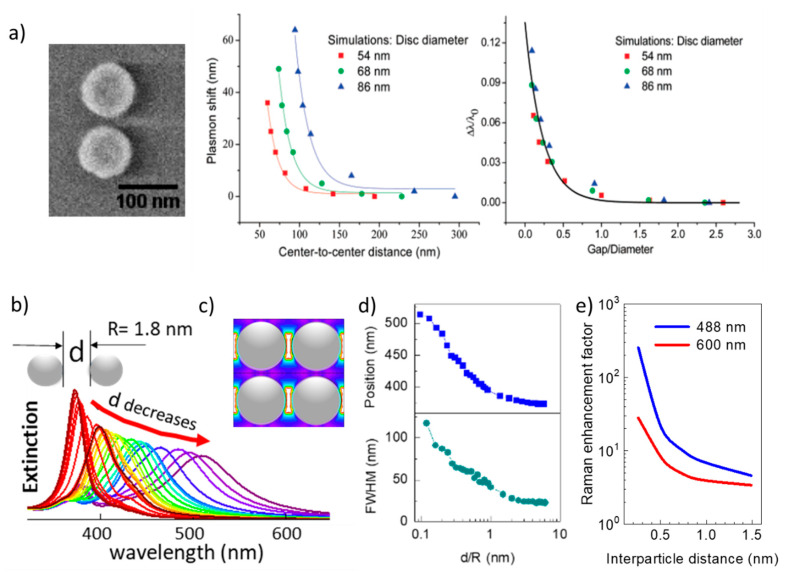
(**a**) Scanning electron microscope (SEM) image of an Au-nanodisk pair and the simulated plasmon wavelength shift vs. the inter-NP distance for three disk sizes using discrete dipole approximation (DDA) and normalized plasmon shift vs. inter-NP distance normalized to the Np diameter (adapted with permission from [46], copyright American Chemical Society, 2007). (**b**) Plasmon for a periodic array of 1.8 nm NPs radius (R) using finite-difference time-domain (FDTD) simulations as a function of the inter-particle distance normalized to R, from d/R = 5 to 0.1 (violet); (**c**) image of the electric field showing the hot-spots; (**d**) position and full width at half maximum (FWHM) of the plasmon in (**b**) as a function of d/R (adapted from [32]); (**e**) EF vs. inter-NP distance for 2 nm radius NPs for two excitation wavelengths.

When two or more NPs are close enough, the electrical field distribution is no longer the addition of the electric fields associated with individual NPs and thus the NPs can no longer be considered as non-interacting. In this case, the particle size that maximizes the enhancement strongly varies depending on the details of the systems. The dependence of the overall enhancement on the inter-NP distance, *d*, is a complex problem since distributions of NPs size and of inter-NPs distances are usually present in real systems, and the formation of hot-spots occurring at the small gaps between adjacent NPs play a very relevant role (Figure 2). The electric field distribution around the NP is even more inhomogeneous when hot-spots are present [43], therefore the electric field at the particular position where the molecule is located relative to the NP can vary tremendously from one location to another (Figure 1d) so that the Raman signal will vary by orders of magnitude. When dealing with relatively high molecule concentrations the effect may be averaged but, for very low analyte concentrations the enhancement depends on the particular location of the tested molecules and the measured Raman signal will vary from one experiment to another. Thus, for extremely low concentrations SERS is intrinsically non-quantitative since it depends strongly on the particular location of the molecules on the NP surface. This tremendous electric field inhomogeneity is especially important for the lowest concertation, the detection of a single-molecule, which relies on hot-spots [44] to obtain the needed EF ~10^9^ [45], and consequently, the experiments are not easily reproducible.

The use of simulations to obtain extinction and Raman amplification are necessary to analyze and understand the processes and foresee optimization directions. Commercial software packages are based on different approximations such as discrete dipole approximation (DDA), boundary element method (BEM), or finite-difference time-domain (FDTD). The discrete dipole approximation is an integral equation solver: particles of arbitrary shapes are modeled by a discrete set of polarizable dipoles, the electric field at each dipole includes that of the rest of the dipoles in the set. FDTD is a differential equation solver. To solve Maxwell differential equations, it uses the finite-difference method in the time domain so that time is also discretized. Solvers using FDTD have been more in use for the last 20 years. The difference between the two methods is mainly operational: how fast a complex system can be modeled and modified, this is probably why FDTD methods have gained more popularity. Also, the dependence on the frequency of the scattering and calculated fields is covered in a single simulation with FDTD methods. The boundary element method (BEM) is also an integral equation solver, it uses integral equation formulation and only needs the values of the fields on the boundary surfaces, the mesh in use is defined on the boundary surfaces and this makes this method less efficient than ordinary FDTD methods for large surface to volume ratios. It is not intended for nonlinear media.

Numerical simulations have to be utilized to include NP-NP interactions; it is then possible to obtain the dependence of the plasmon resonance as a function of the inter-NP distance for different NPs configurations. The calculation for NP pairs, ordered chains, or planes of NP arrays are feasible, but computing time increases dreadfully for large sets of disordered NPs [47,48]. However, state-of-the-art electromagnetic computation techniques can provide clues on the behavior of random arrangements of hundreds of NPs with different shapes.

The variation of the plasmon position was studied in lithographically fabricated pairs of Au nano-disks with inter-particle distances decreasing from 300 to 50 nm (Figure 2a)**.** A red-shift up to 60 nm of the plasmon position as the inter-particle distance is reduced is observed and coincides with simulations using the discrete dipole approximation (DDA) [46]. In the ultra-small limit, the behavior of the simulated extinction spectra was reported for NP radii in the range from 2 to 10 nm when varying the inter-NP distance, *d*, in periodic arrays of silver NPs. The plasmon red-shifts, as in the case of the large Au disks, and also widens as *d* is reduced (Figure 2b). The changes in position and width of the plasmon resonance are significant (position shifts from 355 to >500 nm and width from 25 to >100 nm) and, when plotted against the inter-NP distance normalized to the NP radius, d/R, present an almost identical behavior for radii up to 10 nm (Figure 2d). Such red-shift and widening have been experimentally observed for 4 nm (diameter) randomly dispersed NPs [32]. A general rule is that proximity effects are important when the inter-NP distance is similar or lower than the particle diameter (d ≤ D = 2R). Simulations of NPs ordered arrays describe well randomly spread NPs and have the enormous advantage that the symmetry of periodic distributions allows for much shorter simulation times, but may be insufficient for wide inter-NP distance distributions.

**Figure 3:**
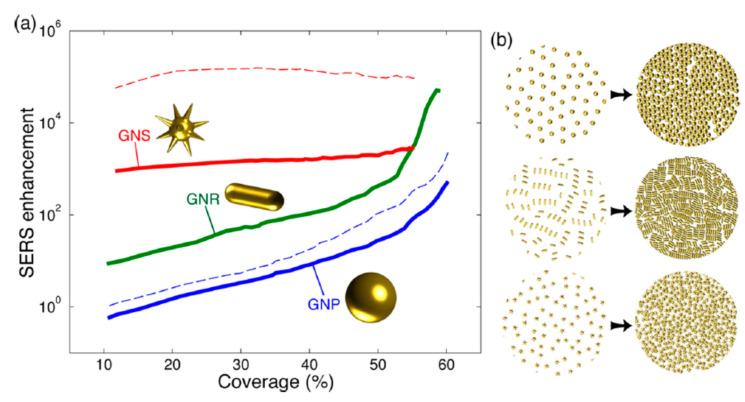
(**a**) Calculated maximum SERS enhancement for different gold particle shapes vs. the coverage (fraction of an NP-monolayer) for 785 nm incident wavelength (solid lines) and (**b**) for a molecule-NP distance of 1 nm. Adapted with permissions from [47].

The relevance of the NP shape and the coverage (fraction of an NP-monolayer on a substrate), which is directly related to the average inter-NP distance, is shown in Figure 3. Solis et al. [47] have used a full-wave solution based on surface-integral equations (SIEs) discretized by the method of moments (MoM) to simulate such SERS platforms with hundreds of NPs. Shape is especially important for non-interacting particles: low coverages/large inter-NP distances. The asymmetry of the NPs and the presence of spikes [49] induce single-NP hot-spots (also called intrinsic electromagnetic hot-spots) that increase the EF [11]. As a practical example, tuning the aspect ratio of silver nanorods, which typically present two resonances, has been used to control the resonance wavelengths and reach the infrared range (related to the long axis of the rods), which is useful for different therapies [50]. For NPs without spikes, the reduction of inter-NP distances (increasing coverage or density) significantly increases the EF due to the formation of hot-spots at the NPs narrow gaps (Figure 3). Note that a coverage of 0.5 corresponds to an average inter-NP distance *d ~ 2R* for spheres. As the inter-particle distance is reduced, the EF increases by orders of magnitude (Figure 2e and Figure 3) overcoming the enhancement related to the hot-spots that originated at the spikes of individual NPs (Figure 3). Thus, at short inter-NP distances, the density of hot-spots is the dominant parameter over size and shape. It is in this regime where the ultra-small NPs (down to a few nm) becomes interesting to be used with visible excitation [32,51].

**Figure 4:**
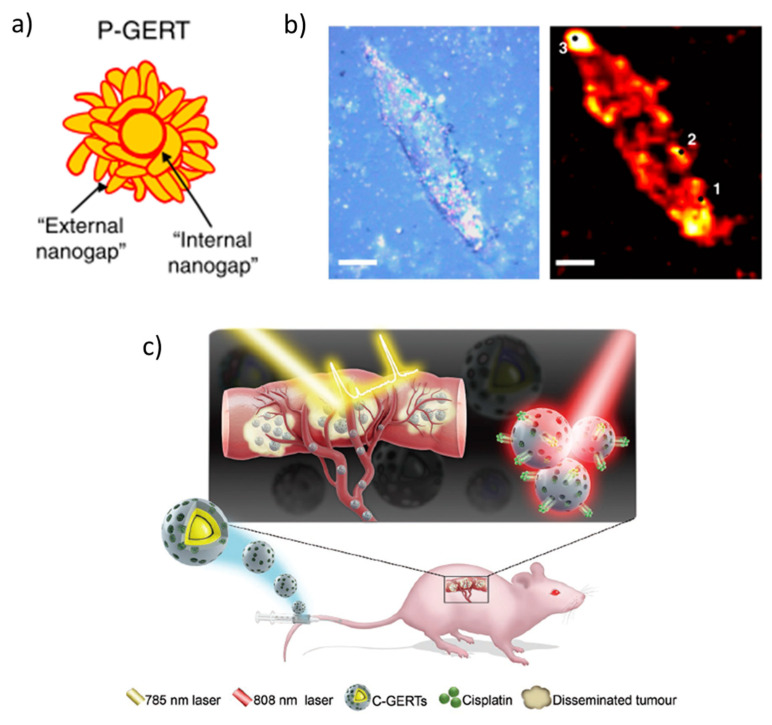
(**a**) Petal-like gap-enhanced Raman tag (GERT), yellow: Au, red: 4-nitrobenzenethiol (4-NBT) Raman reporter with indicated nano-gaps; (**b**) bright-field and Raman (1340 cm^−1^ NO_2_ vibration mode of 4-NBT) images of a single H1299 cell stained with P-GERTs, scale bars are 10 microns. Adapted with permissions from [52]. (**c**) Raman detection of abdominal disseminated microtumors with cisplatin-loaded GERTs. Adapted with permissions from [53]. Copyright John Wiley and Sons, 2018.

Complex core-shell NPs aggregates are designed to generate efficient hot-spots of great interest in biomaterials imaging, so-called gap-enhanced Raman tags (GERTs) (Figure 4a). The cells or tissues are previously stained with petal-like gold NPs aggregates whose morphology is controlled by some Raman reporter, which contains a thiol group to bind the gold NP (such as an aromatic dithiol), forming sub-nanometer internal gaps (red in Figure 4a). In the case of petal-like shell structures with an overall size of around 50 nm, the Au plasmon is red-shifted to ~600 nm and broadened due to plasmonic interactions between the close gold petals [52]. The highly enhanced Raman spectrum of the reporter at the nano-gaps inside the GERTs can be used to detect and image cells and tumors (Figure 4b,c) [53].

Individual plasmonic NPs are, in general, obtained by a chemical route such as the Lee and Meisel’s method [54], can be used as colloids, or can be randomly deposited and immobilized on different solid substrates, depending on the application [10,55,56]. Alternatively, a nanostructured film can be directly deposited on a substrate, typically by physical deposition techniques. An interesting approach to obtain very narrow NP size distributions on substrates is the gas aggregation sputtering technique [16,57,58] that avoids surfactants or any product but is limited to quite small NP sizes (<10 nm). Continuous films deposited on a substrate can be structured by different means such as the nanosphere lithography which is able to produce well-ordered 2D periodic NP arrays [59,60,61]. Indeed, the focus has recently been put on the creation of ordered plasmonic arrays since their optical properties can be tailored beyond size and shape with another parameter, the periodicity of the 2D and 3D structures and hence the inter-particle distance. In that way, plasmon-polariton modes, surface lattice resonance, or extraordinary transmission can be fostered and controlled. Lithographic techniques (such as laser interference, nanosphere, electron-beam, focused-ion, nanoimprint, or anodic aluminum oxide template, among others) offer a wide variety of scales and precision degrees to fabricate 2D and 3D ordered plasmonic structures. The rapid development of the fabrication techniques has endorsed size reduction of the plasmonic objects down to sub-nanometer scale, which are consequently governed by quantum interactions heading to the rise of quantum plasmonics [62].

## 3. Hybrid Graphene-Metallic NPs Systems-The Role of Graphene

Graphene, a one-layer thick carbon lattice formed by sp^2^ C aromatic rings, has an important role to play in different fronts of plasmonics because of its unique electronic, optical, and also mechanical properties. The outstanding properties of graphene are related to its 2D character, the strength of the sp2 covalent bonding, and its hexagonal structure that originates Dirac cones in its electronic structure at the K and K’ non-equivalent points of the reciprocal lattice. The carrier density at the Fermi level of undoped pristine graphene is zero and the mobility of its massless carriers is extremely high (~2 10^5^ cm^2^/Vs). Actual graphene, such as that obtained by chemical vapor deposition (CVD), is always doped, generaly strained and presents defects so that its conductivity and mobility are hindered. However, CVD single-layer graphene still has excellent mechanical and electrical properties with low optical absorption, high specific area, is quite inert, and impermeable to gases, thus offering unique characteristics for optical applications. The combination of graphene with metallic NPs impacts transport properties of graphene [63], the spatial distribution of the NPs’ LSPR, and also may involve charge transfer between metal and graphene thus modifying graphene doping and its Fermi level.

Graphene presents a weakly interacting surface but also allows establishing strong covalent bonds through its functionalization. The use of functionalized graphene, where part of the sp^2^ C=C bonds is broken either by grafting specific functional groups or by using GO, offers interesting possibilities by facilitating the linkage of species of interest. GO can be obtained by chemically exfoliating graphite through oxidation or intercalation and then sonication processes. The oxygen content can be partially tuned by the reduction of GO so that the C/O ratio typically varies from 2 to 6 and the common functional groups are epoxy (C=O), hydroxyl (C–OH), and carboxyl (COOH) [64,65,66]. Carboxyl groups are in general the relevant ones for bio-detection since the anchoring of biomolecules, such as antibodies, is effective only through these groups while the other functional groups of GO worsen electric conductivity and mobility [67]. However, two issues have to be noted: (1) the best reduction processes do not recover the quality of pristine single-layer graphene and (2) the properties of GO and reduced GO totally differ from those of graphene in many aspects besides those related to electric transport. For example, GOs are hydrophilic and easily dispersible in water, contrary to graphene, the chemical-stability and photo-stability are much lower than those of graphene and they are rarely single-layer. While graphene (mechanically exfoliated or CVD) is very robust and is not damaged with radiation densities above 10 MW/cm^2^, defective graphene, graphene nanodots, or graphene oxides are much more photo-sensitive, standing orders of magnitude less light power density. The maximum light density to be used depends strongly on the particular characteristics of the material. Therefore, pristine graphene and the defective variants have, with few exceptions, different applications.

Different configurations have been probed to combine metallic NPs and graphene or GO that here are classified in two groups: those where the NPs are interacting (the NPs are close enough so that their individual electric field distributions are modified and the formation of hot-spots is induced) and those where the NPs are mainly non-interacting (the NPs are far enough so that other NPs do not substantially modify their field distribution). Among the first case are the non-ordered nanostructured metallic films with short average inter-NPs distances obtained by (i) PVD techniques as indicated previously, either directly on graphene or GO or, after deposition can be covered by a graphene/GO layer [1,68,69,70,71]; or (ii) depositing metallic NPs obtained either by chemical routes with wet approaches (spin-coating, doctor Blade, etc.) on the substrate [72,73]. In this case, the generated NPs may have a wide range of sizes and shapes as indicated in the previous section. Alternatively, a continuous flow of ultrafine, chemical-free NPs with controlled size (from 1 to ~20 nm diameter) [74] and narrow particle size distributions [75], is deposited on a substrate by the gas aggregation technique. In the case of non-interacting NPs, there are two main approaches: (i) the wet chemical route that consists in the reduction of metal salts (like HAuCl_4_, AgNO_3_, K_2_PtCl_4_), using reducing agents (like NaBH_4_, and ascorbic acid), and the NP nucleation and simultaneous reduction of GO sheets [8,76] producing suspensions of metallic NPs wrapped by the reduced GO sheets. The size and distribution of synthesized Ag NPs seem to be determined by the strong interactions between the metallic NPs and GO through the controllable reduction of GO [77,78,79]. The second one, (ii), is the nano-structuration, using a wide set of lithographic techniques [62], of a previously deposited metal film producing ordered arrays, and graphene is transferred on top of the created 2D or 3D nanostructures. The elastic properties of graphene provide its adequate conformation onto the NPs.

**Figure 5:**
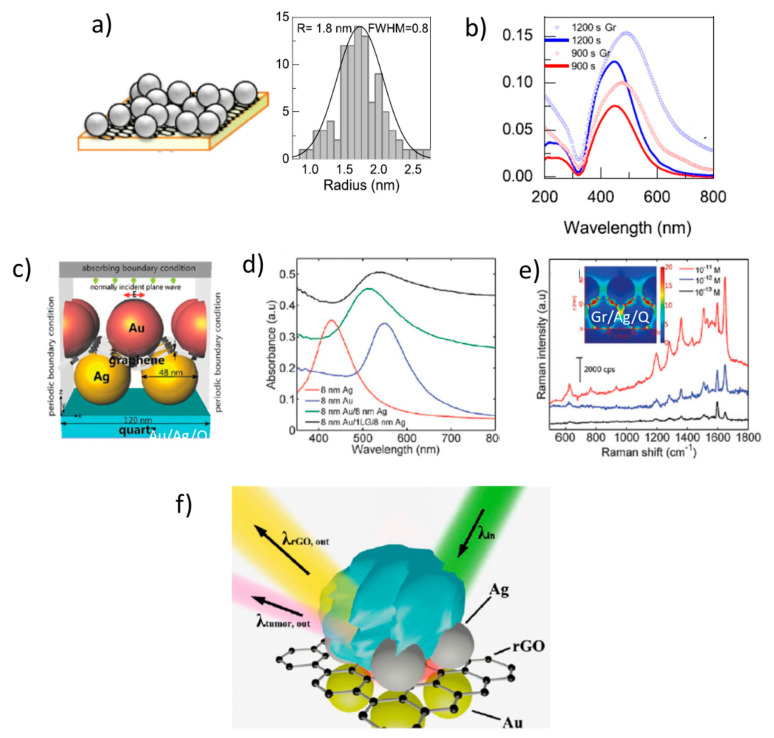
(**a**) Scheme of ultra-small Ag NPs deposited on graphene/quartz substrates and narrow size distribution obtained from transmission electron miscroscope (TEM) images showing an average radius of 1.8 nm; (**b**) absorbance of 1.8 nm NPs with two densities deposited on substrates with (open circles) and without (lines) graphene (adapted from reference [32]); (**c**) Au NPs/graphene/Ag NPs/quartz SERS platforms; (**d**) absorbance of Ag/Au (green) and Ag/graphene/Au (black); (**e**) Raman spectra of RhB on the 8 nm Au/1LG/8 nm Ag/Ag film/quartz for concentrations from 10^−11^ M to 10^−13^ M (adapted with permission of [80], copyright Royal Society of Chemistry, 2014) (**f**) Schema of the proposed mechanism for the tag-free identification and of tumor cells on Ag/rGO/Au SERS platforms. Adapted with permission of [81]. Copyright Springer Nature, 2016.

Graphene significantly influences the plasmon resonance of nanostructured metals as reported for ultra-small Ag NPs deposited on a CVD graphene layer transferred on a transparent substrate or when graphene is intercalated between nanostructured Ag and Au films (Figure 5a,c) [32,80]. In both cases, plasmon absorbance increases, red-shifts, and a wide IR tail appears, probably related to partial electron delocalization promoted by graphene (Figure 5b,d). Combining Ag and Au NPs provides optimized plasmon resonance wavelength for green excitation and the increased Raman activity of Ag versus Au NPs. Moreover, the sub-nm gap in between Au and Ag layers generated by the graphene single-layer allowed extremely low detection limits (Figure 5e). A similar approach of combining Ag and Au films of optimized NP sizes but using reduced GO has demonstrated the capability of identifying tumor cells without using biomarkers (Figure 5f) [81]. Such metal NPs/graphene hybrid systems have applications as SERS platforms [82] and also as transparent electrodes with improved conductivity [17,83,84,85].

**Figure 6:**
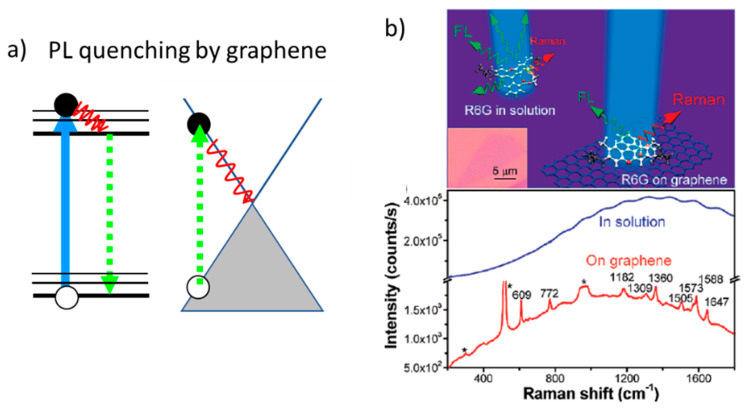
(**a**) Schema of the fluorescence quenching mechanism by graphene. (**b**) Illustration of graphene as a substrate to quench R6G fluorescence and Raman-PL spectra of R6G in water (10 µM) (blue line) and on graphene (red line) at 514 nm excitation; panel (**b**). Adapted with permissions from [92]. Copyright American Chemical Society, 2009.

A chemical enhancement of the Raman signal of several probe molecules associated with graphene was first reported in 2010 [86] and was afterward named GERS (graphene-enhanced Raman scattering). Charge transfer between the molecules and graphene occurs when the Fermi level of graphene is within the HOMO-LUMO gap and the enhancement, as it occurs for other chemical mechanisms, depends on the particular symmetry of the vibration mode and the orientation of the molecule on the substrate. GERS enhancement is reported to be limited to factors in the 10–20 range [87,88] but another factor has to be considered. The high specific area of graphene and GO-based materials increases and facilitates the adsorption of the probe molecules and the π-π interactions occurring with the aromatic rings of the molecules favor their more uniform packing. Therefore, graphene is an excellent bio-compatible platform [89,90,91]. It is especially adequate for biomaterials because of its stability and the weakness of the π-π interactions bypassing the metal-biomaterial interactions occurring in typical SERS platforms. Graphene is also demonstrated to quench molecular fluorescence, especially relevant for the detection of clean Raman signals [92]. Figure 6 presents a schema of the quenching mechanism and an example of R6G fluorescence quenching when deposited on graphene. Fluorescence quenching by graphene occurs by energy transfer from the excited molecule to graphene. Then, the excited electron in the valence band of graphene relaxes to the Fermi level non-radiatively. This process is similar to the Foster resonance energy transfer (FRET) occurring between two molecules (discussed in Section 5). In all cases, the probability of the process strongly decreases as the distance between the molecule and graphene increases.

**Figure 7:**
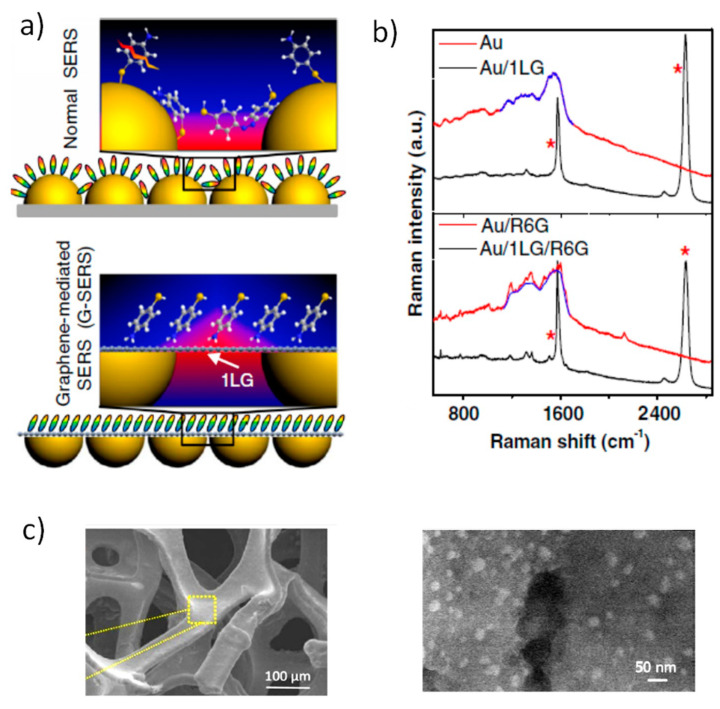
(**a**) Schematic illustration of molecules with different orientations depending on whether they are adsorbed on a normal SERS substrate (gold nanoislands) or on a G-SERS substrate (graphene-coated gold hemispheres); (**b**) shows that the presence of graphene avoids the photo-carbonization of Au (red lines) providing cleaner spectra (black lines). The asterisks indicate the G and 2D peaks of graphene. Adapted from [94]. (**c**) Field enhanced SEM (FESEM) images of graphene foam at low magnification (left) and of AgNPs deposited on graphene foam substrate at high magnification (right). Adapted with permissions from [82]. Copyright Springer Nature, 2016.

Graphene is used as a probe to study the plasmonic system [93], evaluate its amplification capabilities, and detect the formation of hot-spots using its Raman modes: D (1340 cm^−1^), G (1580 cm^−1^), and 2D (2700 cm^−1^) (see black spectra in Figure 7 where G and 2D peaks are indicated with red asterisks) [94]. Moreover, the intensity of graphene D peak, which is activated by the presence of defects, is extremely sensitive to the presence of vacancies, sp^3^ C, functional groups or edges, and small-sized flakes that may be induced during its transfer process from the metallic substrate used for its CVD growth to the SERS system, allowing the estimation of the final quality of the platform. The relative intensity of G and D, ID/IG (and also I2D/IG for low defective graphene) as well as the width of D and G peaks are systematically used to define the quality of graphene and GO [95]. On the other hand, charge transfers between the metal NPs, the probe molecules, and graphene produce a shift of graphene Fermi level. Such doping modifies the position of the G peak towards higher frequencies either for n or p doping, the effect on the 2D peak being smaller. For p doping, the 2D position slightly increases with a relation Δω_2D_/Δω_G_ ~ 0.75 while for n-doping it remains almost constant for low carrier concentrations. Raman phonons of graphene are also sensitive to strain, in this case, the frequencies of G and 2D peaks increase for compressive or decrease for tensile strain with a relation: Δω_2D_/Δω_G_ = 2.2 [96]. Doping and strain contributions can be separated by plotting the 2D peak position against the G peak position in a 2D-G diagram [97]. Therefore, frequency shifts of the G and 2D peaks can detect charge transfer processes occurring either related to the NPs or to the probe molecules.

Graphene is well known to be an excellent barrier against atmospheric gases, it is a one-atom-thick impermeable membrane that protects the metallic NPs from reacting with air and particularly with CO_2_, providing long-term stability to the SERS platforms. In [94] the proposed architecture (Figure 7a, lower panel) avoids the photo-carbonization of Ag and Au NPs (Figure 7b) thus cleaning the related strong background in the 1100–1700 cm^−1^ spectral region where all organic molecules present relevant vibration modes. Graphene has shown the capacity not only to protect the metallic NPs but also to de-oxidize Ag NPs [98]. With the structure proposed in Figure 7a, the graphene layer provides a quite flat surface where the molecules can lean in a uniform way, contrary to the different linking geometries occurring on the NPs, and softens the hot-spots, maintaining the overall amplification capabilities. These characteristics are of interest towards quantitative analyte determination and reproducible results. A different approach to increase the sensitivity of a SERS platform is to increase the surface available for the probe molecules. In this context, multilayer-graphene foams provide an extremely high surface/volume ratio where metallic NPs, and thus analyte molecules, can be deposited (Figure 7c) [82].

**Figure 8:**
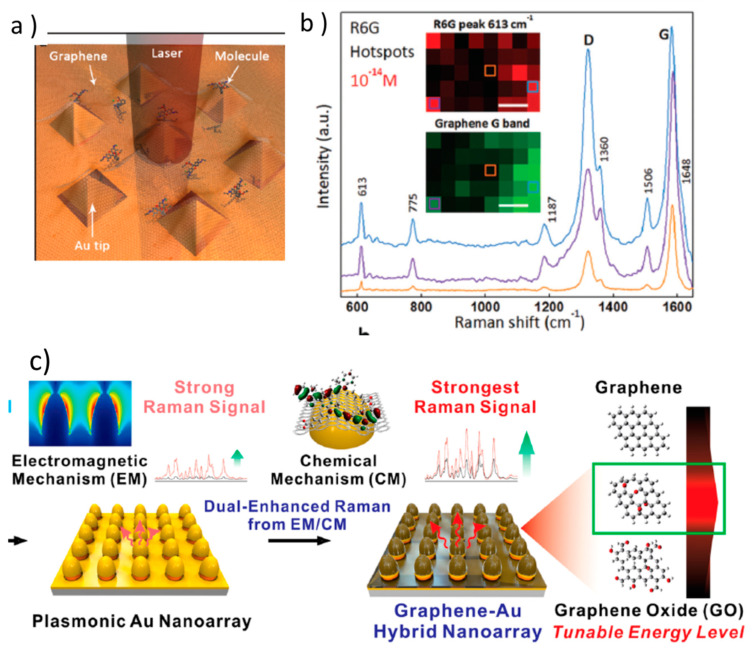
(**a**) Schema of Au nano-pyramids (size around 200 nm) with graphene and deposited molecules; (**b**) Raman spectra graphene and R6G, the insets show Raman intensity images of graphene (G mode) and R6G (613 cm^−1^ band) demonstrating the correspondence of their intensities (scale bar is 2 µm). Adapted with permissions from [99]. Copyright John Wiley and Sons, 2013. (**c**) Array of almost non-interacting Au NPs fabricated by depositing an Au film on a 1 cm^2^ patterned substrate by laser interference lithography. A graphene oxide (GO) layer, with adjusted oxidation, is deposited on top to enhance the CM. Adapted with permissions from [100]. Copyright American Chemical Society, 2019.

Single-molecule detection was demonstrated in non-interacting Au nano-pyramids arrays covered by a transferred CVD graphene single-layer (Figure 8a) [99]. In this case, the intensity variations of graphene Raman spectrum were used to localize the efficient formation of hot-spots, which in this case should be located at the pyramid top, facilitating the detection of the probe molecules (Figure 8b) since only efficient hot-spot areas have to be scanned. This is also an example of how, in real samples, even in ordered lithographed arrays, hot-spots are not uniformly formed (Figure 8b inset). The extreme thinness of graphene does not hamper EM amplification that strongly decreases as the metal-molecule distance increases. Indeed, and extra enhancement due to the GERS chemical mechanism can occur when graphene is included.

Plasmonic non-interacting Au arrays coated with GO have also been demonstrated to produce more uniform and reproducible electromagnetic enhancement (Figure 8c). Moreover, it is possible, by controlling the oxidation degree of GO, to align its energy levels with those of the target molecule thus increasing the CM enhancement and therefore sensitivity [77]. These platforms have been used to detect and quantify a specific biomarker to characterize neuronal differentiation of human neural stem cells [100].

## 4. Interference Raman Enhancement

**Figure 9:**
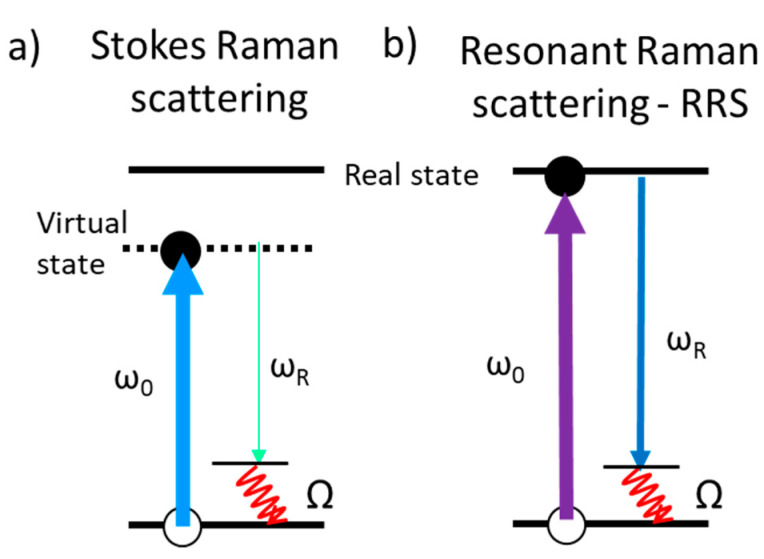
(**a**) Standard Stokes first-order Raman scattering which results in the emission of a phonon/molecule vibration of frequency Ω and a photon of frequency ω_R_ = ω_0_ − Ω with extremely low cross-section; (**b**) resonant Stokes Raman scattering process where the energy of the incident photons coincides with a real electronic transition, resulting in a large Raman cross-section increase.

Besides the electromagnetic enhancement due to localized surface plasmon resonances of metallic NPs and the chemical enhancement related to charge transfer or modifications of the molecular electronic structure described in the previous sections, other mechanisms are investigated as the use of excitation wavelengths in resonant conditions for the probe molecule (resonant Raman scattering, or RRS, Figure 9), that is, when the laser wavelength coincides with absorption maxima. In this condition, usually in the UV for many molecules, Raman cross-section can increase orders of magnitude. If the plasmon resonance is also located in the UV spectral region then RRS and SERS effects can be combined. This possibility has promoted the interest towards UV-SERS thus searching NPs with efficient plasmon resonances in the UV [101].

An alternative mechanism is light interference that can be exploited to enhance Raman and PL (fluorescence and phosphorescence) signals by adequately designing multilayered platforms. Interference of light associated with multireflections at material interfaces is well known and used for a large number of applications, such as selective filters or coatings. In 1980 Raman gains up to 20 were reported for Ti, Ti_2_O_3_, or Te ultra-thin films by interference-enhanced Raman scattering (IERS) [26,27]. Serendipity promoted the extended use of SiO_2_/Si substrates to deposit and study graphene since a particular SiO_2_ thickness range permits increasing the contrast in optical images allowing the detection of single-layer graphene, with an absorption in the visible range of 2.3%, which is not identifiable on bare silicon. This method is of special interest to localize the micron-sized mechanically exfoliated graphene flakes and provides Raman amplification factors up to 30–40 [28,29,30,102].

**Figure 10:**
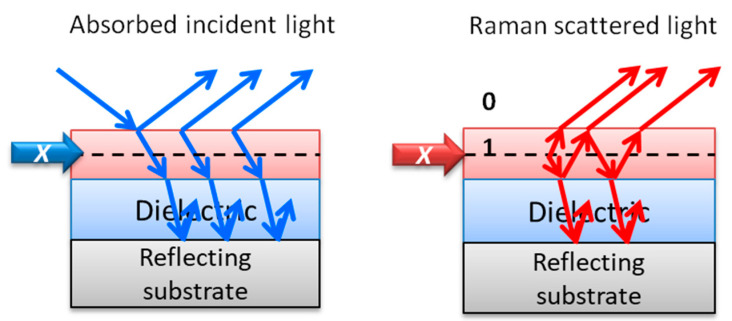
Schemas for the interference of incident and scattered light for the simplest interference platform. The layer where the enhancement is calculated is named 1 and 0 corresponds to air.

Interference requires flat and parallel interfaces limiting materials with different refraction indices. The simplest system consists of a highly reflecting substrate and a dielectric layer. The constructive interference can be maximized for a given wavelength varying the thickness of the dielectric and the refractive indices of all involved media. To optimize Raman interference amplification from a multilayered structure, graphene phonons G and 2D can be used as an adequate probe. Figure 10 presents a schema of the interference processes for the simplest system: reflecting layer/dielectric layer/graphene. The propagation of light through such a multilayer can be calculated using the transfer matrix method obtaining separately the absorbed and scattered lights at each point of the medium of interest (layer 1 = graphene) by using matrices that relate neighboring layers by the product of two matrices. To calculate the effect of multiple reflections on the Raman intensity measured from layer 1 it is necessary to calculate the absorbed amplitude, *Fab*, of the waves at a depth x in layer 1, if light of amplitude *I_0_* = 1 is impinging from medium 0. Next, the scattered amplitude, *Fsc*, of the waves that leave the film back to medium 0 as a consequence of the scattering processes at depth *×* in layer 1 has to be calculated. The resulting Raman intensity (*I*) is proportional to:

(1) $$I\sim \int_{0}^{d}{\left|F_{ab}F_{sc}\right|}^{2}dx$$

where the integral is over the thickness of layer 1 and *F_ab_* and *F_sc_* are obtained using the matrix transfer method [103].

**Figure 11:**
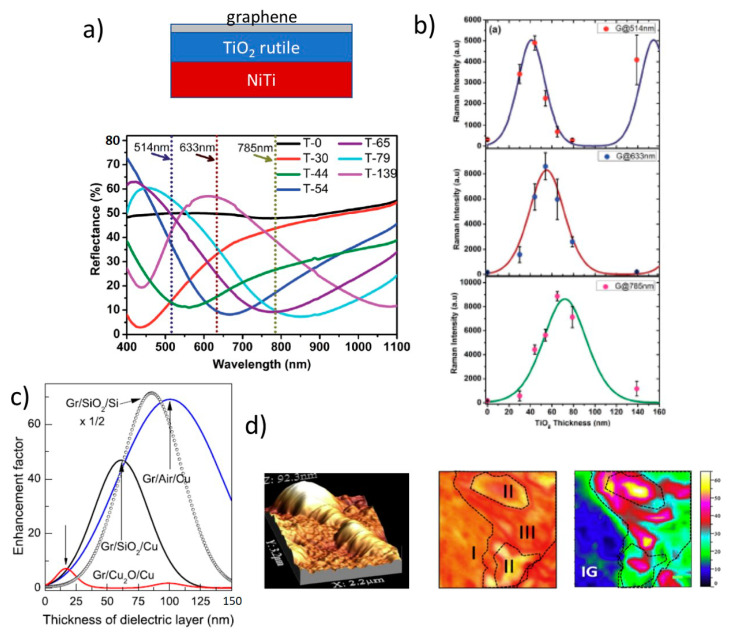
(**a**) Scheme of the graphene/TiO_2_/NiTi interference system and the measured reflectance as a function of the TiO_2_ oxidation time/layer thickness; (**b**) G Raman peak intensity measured and calculated for three excitation wavelengths vs. the TiO_2_ thickness. Adapted with permission of [105], Copyright Royal Society of Chemistry, 2016. (**c**) Calculated enhancement factors (EF) for graphene G peak on Cu_2_O/Cu, SiO_2_/Cu, air/Cu, and SiO_2_/Si; (**d**) Atomic force microscopy (AFM) image of a graphene bubble on copper and the optical and G Raman peak Intensity images of a region with several bubbles. Adapted with permission of [107]. Copyright Elsevier, 2016.

Recently, enhancements around 80 have been reported for F_16_CuPc on Si/SiO_2_ with an optimized SiO_2_ thickness of 90 nm [104], and around 50 for NiTi/TiO_2_/graphene heterostructures [105] where the TiO_2_ layer is formed by the controlled oxidation of the NiTi substrate. Figure 11 shows the reflectivity (in b) of the multilayer (in a) for different oxidation times (thus different TiO_2_ thickness), showing oscillations due to interference of the multiple reflections at the different interfaces and, (in c), the large variations of the measured and calculated Raman intensities of graphene G peak for three excitation wavelengths (514, 633, and 785 nm) as a function of the TiO_2_ thickness. Low and real refraction index for the dielectric layer is in principle preferred, however, graphene Raman signal is also amplified (5–7 fold) by copper oxide thin layers (*n* = 3.121 + *i* 0.295, at 500 nm) in Cu/CuO_2_/graphene [106] but the signal is boosted in graphene bubbles on copper where the dielectric is the encapsulated gas (*n* = 1) [107] (Figure 11c) reaching EF up to 70.

**Figure 12:**
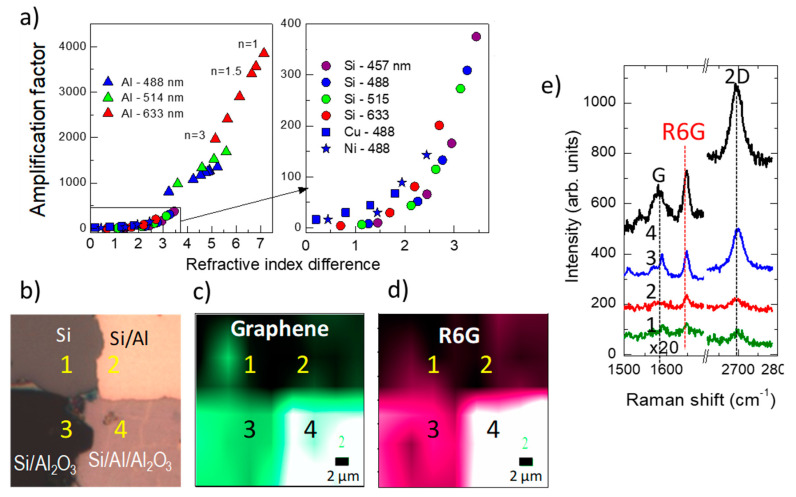
(**a**) Amplification factor of G intensity for different graphene/dielectric/reflecting layer (Al (triangles), Si (circles), Cu (squares) and Ni (stars)) systems at 633 nm (red symbols), 514 nm (green), 488 nm (blue), and 457 nm (purple), laser excitation as a function of |n(dielectric) –n(reflector)|. For each reflecting material, several values of n(dielectric) ranging from 1 to 3 are calculated. (**b**) Optical image (20 × 20 µm) of the four zones of the heterostructure with 70 nm Al_2_O_3_ with transferred graphene and spin-coated with R6G. Raman images of the same region of (**c**) the 2D graphene peak and (**d**) the R6G 1645 cm^−1^ peak. (**e**) Representative Raman spectra of each of the four zones. Adapted with permissions from [103]. Copyright American Chemical Society, 2017.

A proposed architecture, Si/Ag/Al_2_O_3_/graphene, includes silver as a reflecting layer and also as a plasmonic component to promote combined SERS and IERS amplification of the graphene signal, however with low EF ~ 20 [108]. Calculations to optimize IERS in reflecting/dielectric/graphene heterostructures indicate that the best situation occurs when the imaginary part of the reflecting layer refractive index is maximized and the real part for the dielectric layer is one. A universal correlation is found for the calculated EF (corresponding to the optimum dielectric thickness in each case) and the modulus of the complex refractive indices difference |Δ*n*| = |*n(dielectric) – n(reflector)*| for all tested reflecting layers (Cu, Ni, Al, and Si) and for dielectric layers with a refractive index between 1 and 3 using typical excitation wavelengths in Raman spectroscopy (457, 488, 514, and 633 nm) (Figure 12a). It is concluded that the best experimentally easily accessible heterostructure is the combination of an aluminum film deposited on the extremely flat surface of a silicon wafer and a low-*n* oxide film, such as Al_2_O_3_ or SiO_2_. The calculated EF for Si/Al (90 nm)/Al_2_O_3_ (60 nm)/graphene system reaches 1200 for excitation at 488 nm and 10^4^ for 1000 nm. The experimental EF at 488 nm is reported to be around 700 for the Raman peaks of the transferred graphene and slightly below for R6G Raman modes which were spin-coated on top (Figure 12b) [103].

In order to increase the EF, some studies report on the possibility to combine IERS and SERS mechanisms, [108] finding, however, relatively low amplification factors except when Ag NPs are deposited on top of Si/Al/Al_2_O_3_/Gr platforms where the combined enhancement is shown to be very efficient [103]. Other architectures have been recently proposed to enhance the Raman signal by increasing the specific area and thus the surface available for the molecules to be adsorbed, for example, by the combination of nano or micro-structured semiconductors (nanopillars, nanorods, etc.), with metallic NPs [109,110] or the formation of nanoporous metals [111].

**Figure 13:**
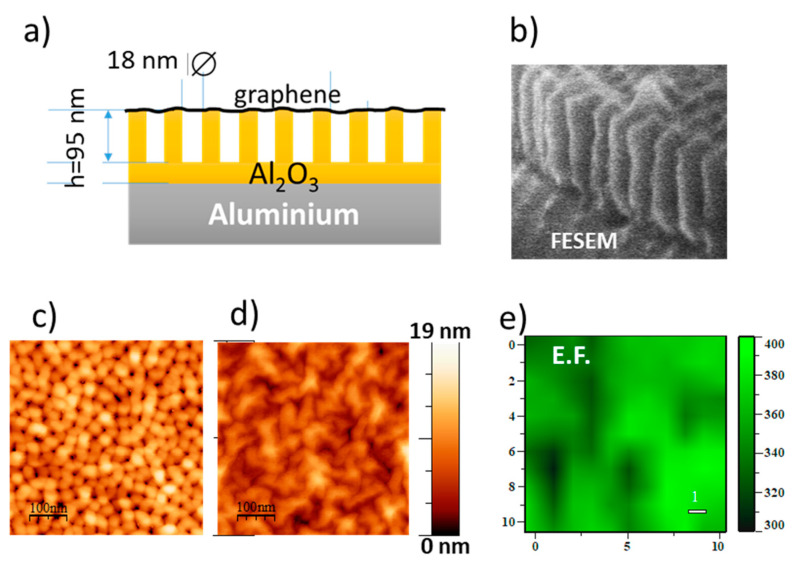
(**a**) Schema of the section of a supported alumina membrane with single-layer graphene transferred on top of it. (**b**) SEM image of the alumina supported membrane with h ≈ 200 nm. AFM topographic images of (**c**) the pristine membrane and (**d**) after graphene transfer. (**e**) Image of the enhancement factor EF = I_2D_(membrane)/I_2D_ (fused silica) of 2D peak of graphene on a h = 100 nm membrane. Adapted from [112].

Graphene allows designing novel architectures such as interference platforms based on supported porous alumina membranes and graphene. Alumina and voids play the role of the dielectric layer, the aluminum foil at the base is the reflecting surface, and a transferred graphene single-layer on top serves to deposit the analyte on the voids (Figure 13). IERS with EF up to 400 and homogeneous amplification over the sample surface are obtained (Figure 13e) [112].

## 5. Fluorescence Enhancement and Imaging

**Figure 14:**
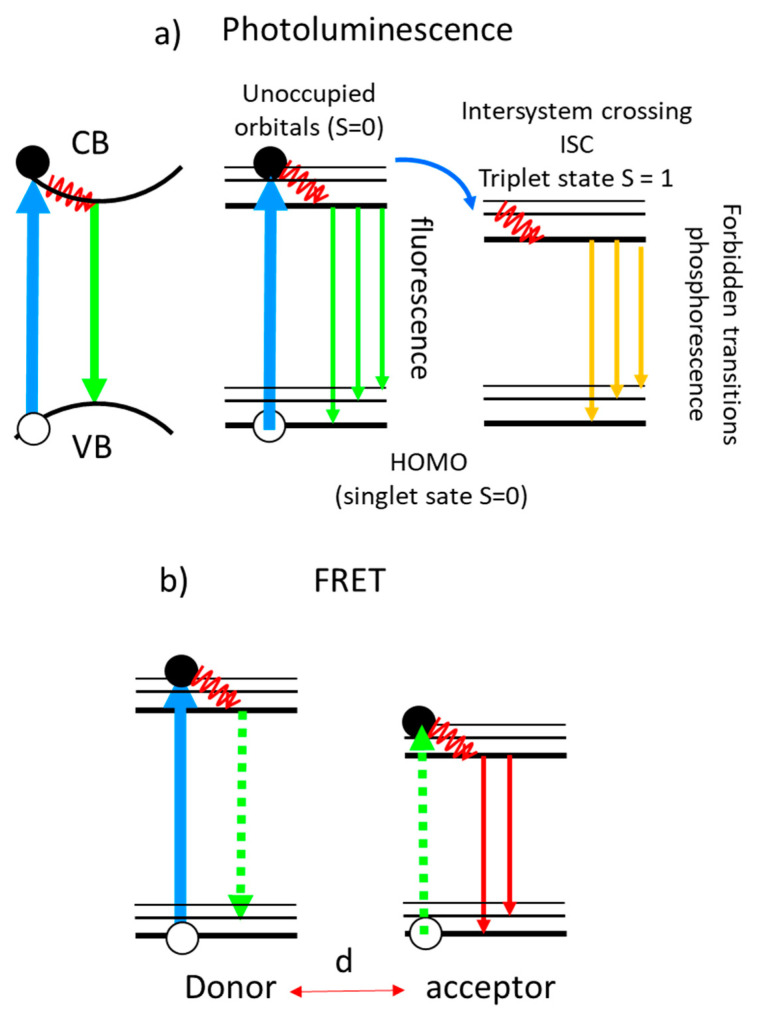
(**a**) Photoluminescence process in a solid (VB = valence band, CB = conduction band, no excitonic effects are considered); fluorescence and phosphorescence processes in a molecule (HOMO = highest occupied molecular orbital, typically with S = 0). The red waves indicate phonon/vibration and vibrational relaxation; (**b**) fluorescence after an energy transfer from a donor molecule to an acceptor molecule. The dashed vertical arrows are virtual photons.

There is a huge interest in enhancing PL signals, especially those originated from markers in biomaterials, to reduce the detection limits and to improve optical imaging. Simplified schemas of PL processes are presented in Figure 14. The first schema corresponds to the conduction to valence band emission in a solid without exciton formation. The other schemas correspond to luminescent molecules (also called chromophores, fluorophores or dyes) using the Jablonski diagrams where fluorescence and phosphorescence processes are shown (Figure 14a). After light absorption, and electron transition from the fundamental state with S = 0 (singlet) to an excited electronic (also with S = 0 since ΔS = 0 in an optical transition), the electron relaxes to the lowest vibrational state (red wavy line in Figure 14) of this excited electronic state (vibrational relaxation) and then radiatively decays to the fundamental state (fluorescence). However, molecules with high spin-orbit coupling have a higher probability to relax to an excited triplet state (S = 1) through the intersystem crossing mechanism (ISC). The radiative transition (phosphorescence) is spin forbidden so the lifetime of the triplet excited state is long as well as the lifetime of the emitted photons.

Important efforts have been devoted to study and optimize light enhancement using metallic substrates and NPs, also called metal-enhanced fluorescence (MEF) or surface-enhanced fluorescence (SEF), but the process is not totally understood and is many orders of magnitude less efficient than SERS. Enhancement factors below 15, typically, are reported for fluorescence both from experiments and calculations [113]. In many cases, the observed PL enhancement is mostly due to an increase in the absorption rates provided by the NPs that are acting as antennas rather than the modification of the spontaneous emission rates. The emission rate is related to the coupling of the emitter with the metal NP that involves many parameters such as the radiative and non-radiative decay rates of the emitter and of the NP, the quenching of the emitter by the metal, and the decay of the emitter by exciting plasmons [114]. Moreover, the interaction processes are strongly dependent on the emitter location relative to the NPs [115,116]. A reduction of the emission measured lifetime is usually observed due to new non-radiative de-excitation channels provided by the metal so that the fluorescent molecules have to be separated from the NPs by several nanometers to avoid total quenching. Fluorescence quenching by metal NPs is similar to that for graphene (Figure 6b). The process occurs by an energy transfer from the molecule to the metal without the participation of photons (the dashed vertical lines are virtual photons). This is a non-radiative process and is very similar to the Fluorescence or Förster Resonant Energy Transfer (FRET) described in Figure 14b between two molecules. FRET is due to a dipole-dipole interaction with a strong dependence on the distance between donor and acceptor (~1/d^6^) and on their spectral overlap (emission of the donor and absorption of the acceptor). FRET thus can occur for donor-acceptor distances typically below 10 nm [117]. Radiative transfer processes involve the emission of a photon that is afterward absorbed by an acceptor. FRET is commonly used to monitor biological reactions that occur at close distances by analyzing the fluorescence quenching induced by the involved processes.

Even if not comparable to SERS enhancement values, metal-enhanced fluorescence does improve signal detectability, increases intrinsic DNA fluorescence, and reduces blinking of single molecules [118]. A wide variety of fluorophores with different application fields have been tested. The fluorescence of individual CdTe quantum dots coated with PVA increases 5-fold when deposited on silver island films [14]. Metallic nanoporous Al-Mg alloys can be exploited for fluorescence enhancement in the deep UV [23]. Indeed, aluminum has been predicted to present the maximum plasmonic performance for UV [25].

Graphene and GO do not present fluorescence but have a role in fluorescence enhancement while graphene and GO quantum dots or nanodots (GQD), as well as carbon nanodots (CD), can produce efficient fluorescence emission, fostering interesting applications in biosensing. These graphene-related materials are adequate for theranostics by enabling fluorescence imaging combined with different therapeutic modalities [21,119]. Hereafter several applications of graphene derivatives are presented as examples of their convenience in bio-detection and imaging.

Among the variety of applications, graphene-related materials are attractive alternatives in the promising photodynamic therapy (PDT). Photodynamic therapy is based on the generation of highly reactive singlet oxygen by light irradiation of an injected photosensitizer to destroy target cells. The photosensitizers (PS) facilitate the fluorescence imaging and the time evolution upon light irradiation of the tissues modified by the generated singlet oxygen [120]. A variety of photosensitizers are used for photodynamic therapy, as liposomes and NPs of different nature (polymer, silica, magnetic, or gold), but their limited water solubility or fluorescence quenching are serious issues. Diverse strategies are being investigated to overcome these problems.

**Figure 15:**
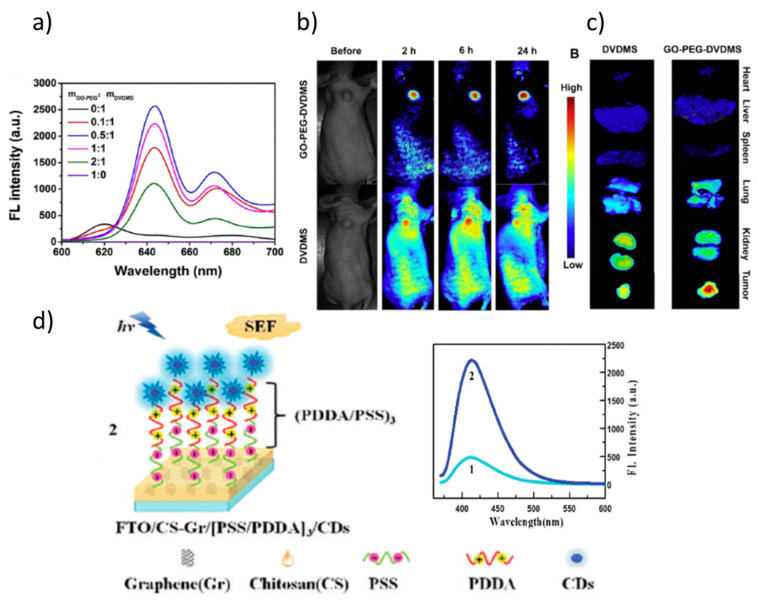
(**a**) Fluorescence spectra of GO-PEG-DVDMS at different weight ratios of GO-PEG: DVDMS; (**b**) in vivo distributions of GO-PEG-DVDMS and DVDMS visualized by using a molecular imaging system before and 2, 6, and 24 h after intravenous administration (DVDMS 2 mg/kg); (**c**) ex vivo near-infrared (NIR) fluorescence images of a tumor and major organs collected at 24 h after DVDMS or GO-PEG-DVDMS injection. Adapted with permissions from [121]. Copyright Elsevier, 2015. (**d**) Illustration of the (2) FTO/CS–Gr/[PSS/PDDA]_3_/CDs system used to enhance the CDs fluorescence and fluorescence spectra of the CDs with (2) and without (1) the reduced GO layer (CS-Gr) for the optimized poly(diallyldimethylammonium chloride)/ poly(sodium styrene sulfonate (PDDA/PSS) layer thickness. Adapted with permission from [122]. Copyright Elsevier, 2015.

Regarding the role of GO, in many cases, when used as a nanocarrier of the photosensitizer, it produces fluorescence quenching. However, it can also promote and enhance fluorescence as in the developed nanoplatform based on GO -amine-terminated eight-arm branched PEG-DVDMS (photo-theranostic agent based on sinoporphyrin sodium). GO-PEG was demonstrated to enhanced the fluorescence of DVDMS. The emission is red-shifted and significantly enhanced, finding the maximum fluorescence yield for a relation 0.5:1 between GO-PEG and DVDMS (Figure 15a). These modifications are due to an intramolecular charge transfer within DVDMS mediated by its coupling to GO-PEG. The increased emission yield permits effective enhanced fluorescence imaging-guided photodynamic therapy for diagnosis and treatment of cancer (Figure 15b,c) [121].

On their side, GQDs and CDs present efficient fluorescence associated with their nanometric size that can be tuned by modifying size and oxygen content. They are better suited for biological applications than most semiconductor quantum dots that contain heavy metal elements. GQDs are nanosized flakes (typically <10 nm) of few-layer graphene or graphene oxide [123] while CDs are almost spherical NPs (<10 nm) of sp2 C atoms that, mainly at the surface, present sp3 C linked to COOH and OH groups [124]. Both GQD and CD present remarkable photoluminescence (PL) properties, high water solubility, chemical inertness, and resistance to photobleaching. The functional groups present at the GQDs’ and CDs’ surface can be easily functionalized, are relatively stable, present tunable luminescence and low cytotoxicity, and thus good biocompatibility. The PL is due to bandgap transitions but their nature is still unclear [125]. An example of the combination of two uses of graphene derivatives is shown in Figure 15d. An enhancement of the fluorescence intensity of CDs has been demonstrated to be originated by the presence of a reduced GO layer, therefore without including metal NPs in the system (Figure 15d). The distance between the emitters (CDs) and the reduced GO layer was varied and optimized to maximize the CDs fluoresce quantum yield [122].

**Figure 16:**
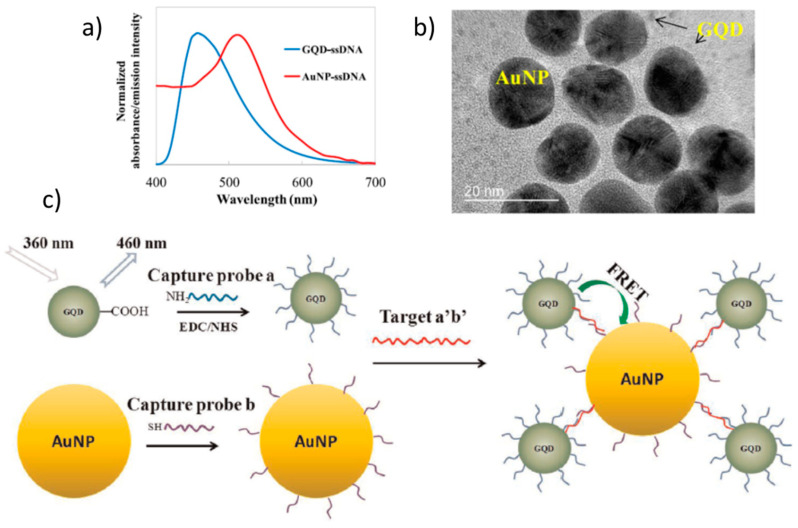
(**a**) Overlap of donor GO quantum dots (GQD) emission and acceptor Au NPs absorption; (**b**) TEM images of conjugated AuNP (~15 nm) and GQDs (~3 nm); (**c**) Schema of the sensing mechanisms of GQDs-AuNPs Foster resonance energy transfer (FRET) biosensor for S. aureus gene detection. Adapted with permissions from [127]. Copyright Elsevier, 2015.

Graphene-related materials have also recently triggered studies on the possibilities they open for FRET applications (FRET mechanism is pictured in Figure 14b). FRET is extensively used in biosensing, in particular, to follow biological reactions that are occurring at close distances, such as antibody immunological recognition or DNA hybridization, by studying the fluorescence quenching occurring when donor and acceptor are close enough. So adequate and optimized donors and acceptors can enhance the sensitivity of FRET biosensors. Typically, fluorescent organic molecules and proteins have been used but their stability is poor and their photobleaching easy. Inorganic quantum dots and more recently graphene-related materials are being investigated [126]. Figure 16a shows that the spectral overlap of GQDs PL spectra and AuNPs absorption is adequate for FRET. When the target S. aureus gene oligo, formed by the union of amine-modified capture probes and reporter probes, becomes linked, the donor-acceptor distance is reduced and the quenching of the GQDs occurs (Figure 16c) [127].

These few examples are intended to show that graphene and its derivatives are in the focus for bioimaging and therapeutic applications however, their toxicity has to be carefully evaluated to be transferable to in vivo applications. Toxicity depends on several factors such as their size, shape, surface chemistry, and solubility since their physicochemical interactions and accumulation in specific organs vary depending on these properties [20,21,128]. A determining factor is the dose concentration, for example, in in-vitro experiments a dose concentration of 200 µg/mL of GO causes oxidative stress, inducing a loss of cell viability. However, low GO concentrations (10 µg/mL) do not enter the cells and have no obvious toxicity [129]. The impact on the toxicity of the size of the GO flakes has also been studied, finding that the smallest GO showed the highest hemolytic activity [130]. Toxicity was also studied by modifying reduced GO. Hydrophobic reduced GO tends to accumulate on the cell membrane in large amounts, inducing high reactive oxygen species stress which leads to cell apoptosis but, after functionalizing it with carboxyl groups to make it hydrophilic the toxicity was largely eliminated, indicating that water solubility may also influence the toxicity of reduced GO [131]. Surface modifications of GO and reduced GO with polymers are also possible solutions to the toxicity problem. For example, the hemolysis of GO obtained by sonication was significantly reduced with chitosan coating on its surface [130] and no cytotoxicity is found for GO after being modified with PEG [132].

Among other different approaches for fluorescence enhancement, the plasmonic nanocavities and the interference-based platforms are at initial development stages and graphene has been scarcely incorporated. The plasmonic nanocavities are usually formed at the gaps between a metallic surface and metallic NPs. The metallic surface, the NPs, or both are coated with a dielectric layer to avoid electric contact and the delocalization of the plasmons. This insulating layer is also required to avoid fluorescence quenching [116]. Complex experimental systems and mathematical treatments are being developed for single-molecule optical studies using plasmonic nanocavities [133,134] that allow nanometric spatial resolution, well below the theoretical diffraction limit for visible light (around 200 nm for 500 nm excitation wavelength).

**Figure 17:**
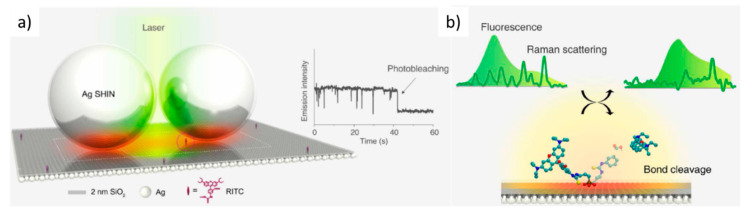
(**a**) Schema for plasmon-enhanced single-molecule spectroscopy in a plasmonic nanocavity. Right inset: emission intensity blinking behavior of a single-molecule and a single-step photobleaching event. (**b**) Real-time detection of the photo-induced cleavage reaction of a surface rhodamine B isothiocyanate (RITC) molecule by correlating simultaneous fluorescence and Raman signals. Adapted from [135].

Recently, single-molecule modifications have been followed in real-time using a standard confocal Raman system and a plasmonic cavity formed by a silver surface coated with a 2 nm SiO_2_ layer and silver shell-isolated NPs (~60 nm Ag diameter) (Figure 17a) [135]. The photo-induced bond cleavage between the xanthene and phenyl group of a single rhodamine B isothiocyanate molecule is studied by combining Raman and fluorescence signals (Figure 17b). In addition, recently enhancements of the intensity of graphene Raman phonons and of graphene absorption have been reported when located in plasmonic nanocavities [136,137]. 

**Figure 18:**
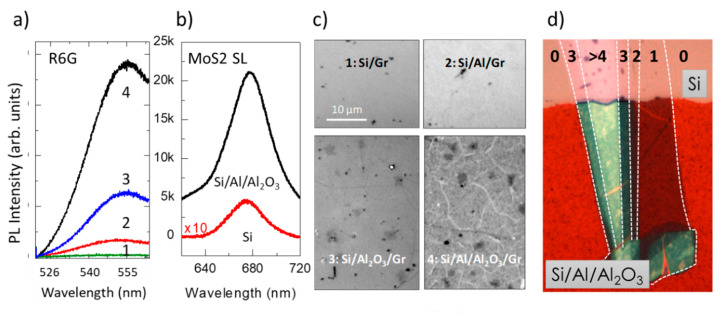
Fluorescence interference enhancement of (**a**) R6G (#1 to #4 correspond to the four zones in (**c**)) and (**b**) single-layer MoS_2_. Optical images of (**c**) single-layer graphene (adapted with permission from [103], copyright American Chemical Society, 2017), and (**d**) MoS_2_ exfoliated flake, on interference platforms.

While the processes leading to Raman and PL enhancements in the vicinity of metallic NPs are different and generate very different EF; the interference mechanism is identical for Raman and fluorescence except that there is often a larger wavelength difference between the incident and scattered beams in the latter case. This detail has not an important impact since the refractive index variation on the typical wavelength ranges is usually moderate for the involved materials. However, scarce works have reported on the use of light interference for fluorescence enhancement but such platforms may be of interest for fluorescence bioimaging. EF as high as 100 was observed for R6G fluorescence signals (Figure 18a) when deposited on a Si/Al/Al_2_O_3_ heterostructure without graphene thus avoiding fluorescence quenching. Large enhancement factors, around 40 for Raman and 50 for PL, were also detected for single-layer MoS_2_ in a similar Si/Al/Al_2_O_3_ platform (Figure 18b). Moreover, these platforms strongly enhance the quality of the white light optical images. The wrinkles, two-layer micron-sized areas, and other defects in single-layer graphene obtained by CVD and transferred to such platforms are clearly seen (Figure 18c). In Figure 18d, single-layer MoS_2_, is clearly visualized on the platform (lower part of the image) and the regions with 2, 3 and 4 layers are well differentiated. On the contrary, MoS2 is almost undetectable (for 1and 2-layer MoS_2_) on silicon (upper part).

## 6. Summary and Forecasting

Graphene, graphene foams, and functionalized graphene (such as GO with different reduction degrees) have a primordial role in novel architectures of SERS platforms to enhance both electric and chemical enhancement mechanisms, to reach single-molecule detection, to stabilize and gain reproducibility, to homogenize the electromagnetic fields when quantification is focused, and to increase the specific area where the probe molecules can be deposited or adsorbed. Finally, graphene can quench undesired fluorescence of the probe molecules and avoids the photo-carbonization of metal NPs thus allowing specific sensing by cleaning up backgrounds in Raman spectra. While pristine graphene offers unique possibilities in the design of enhancement platforms due to its extreme thinness, mechanical properties, and chemical inertness, functionalized graphene and the several variants of graphene and carbon nanodots are priceless for biosensing and bioimaging.

The design of plasmonic cavities has great potential for further developments in the field. While graphene has not yet found a relevant role to play in such systems, other 2D materials, with low conductivity, may be of interest to generate ultra-narrow gaps. The design of novel architectures favoring homogeneous and reproducible enhancements is still required to reach reliable quantification of analytes and for adequate imaging.

Other enhancement mechanisms of Raman signal such as resonant and interference Raman scattering (RRS and IRS), as well as their integration in SERS platforms, can be further developed. However, both have their specific limitations. RRS can produce enhancements of several orders of magnitude, but the high absorption by the probe molecules at the incident laser energy can provoke their rapid photobleaching. Therefore, an appropriate balance between amplification and stability has to be found. IERS can be combined with SERS but the density of deposited NPs has to be relatively low.

The enhancement of fluorescence signals is a very relevant objective in bio-detection, for example, to reduce the threshold limit in the detection of cancer biomarkers, hormones, allergens, or proteins, and in bioimaging using fluorescence markers. While fluorescence is an extremely sensitive technique, improving sensitivity for single-molecule detection requires the development of new designs and approaches. Indeed, plasmonic enhancement of fluorescence still has low efficiency due to the compromise imposed by metal-induced quenching. Graphene and carbon nanodots seem to have an important role to play due to their biocompatibility and much higher stability compared to organic dyes. However, the in vivo biotoxicity of graphene and related nanomaterials has to be further investigated. Finally, interference enhancement-based platforms with uniform and controlled amplification could bring the possibility of obtaining optical images with enhanced sensitivity using either the intensity of a Raman peak of the molecule to be tested, a fluorescence signal, or the white light of a microscope with applications in bioimaging and detection.
